# Autologous Blood Injection and Wrist Immobilisation for Chronic Lateral Epicondylitis

**DOI:** 10.1155/2012/387829

**Published:** 2012-12-04

**Authors:** Nicola Massy-Westropp, Stuart Simmonds, Suzanne Caragianis, Andrew Potter

**Affiliations:** ^1^School of Health Sciences, University of South Australia, Adelaide, SA 5000, Australia; ^2^SA Hand Therapy, Goodwood, SA 5035, Australia; ^3^Ashford Specialist Centre, Ashford, SA 5035, Australia

## Abstract

*Purpose*. This study explored the effect of autologous blood injection (with ultrasound guidance) to the elbows of patients who had radiologically assessed degeneration of the origin of extensor carpi radialis brevis and failed cortisone injection/s to the lateral epicondylitis. *Methods*. This prospective longitudinal series involved preinjection assessment of pain, grip strength, and function, using the patient-rated tennis elbow evaluation. Patients were injected with blood from the contralateral limb and then wore a customised wrist support for five days, after which they commenced a stretching, strengthening, and massage programme with an occupational therapist. These patients were assessed after six months and then finally between 18 months and five years after injection, using the patient-rated tennis elbow evaluation. *Results*. Thirty-eight of 40 patients completed the study, showing significant improvement in pain; the worst pain decreased by two to five points out of a 10-point visual analogue for pain. Self-perceived function improved by 11–25 points out of 100. Women showed significant increase in grip, but men did not. *Conclusions*. Autologous blood injection improved pain and function in a worker's compensation cohort of patients with chronic lateral epicondylitis, who had not had relief with cortisone injection.

## 1. Introduction

Lateral epicondylitis or tennis elbow is a common condition that causes pain on the outside of the elbow, as well as pain and weakness during gripping. It has been found to occur in approximately 1.3% of people in studied populations [1]. Tennis elbow is commonly associated with obesity, smoking, and physical loading during activity, as well as playing tennis [1]. The site of long-term scarring has been shown (during ultrasound) to be where the extensor carpi radialis brevis muscle, which lifts the wrist, originates from the humerus [2].

There are many conservative treatments, including splinting, massage, injection of nonsteroidal anti-inflammatories, and alteration of tasks performed by the patient. There is high level, high quality evidence to suggest that extracorporeal shock wave therapy has little or no benefit [3] and that the evidence for orthotics and splints is not clear [4], but a high number of studies suggest that injection of nonsteroidal anti-inflammatories provides good immediate pain relief, with variable recurrence rates of symptoms [4]. Corticosteroid injection has been shown to provide short-term relief but relapse rates are high [5].

Autologous blood injection (ABI) is theorized to stimulate a “healing cascade” of events, in the degenerated tendinous origin of extensor carpi radialis brevis [6]. Two to three millilitres of the patient's blood is removed from their contralateral arm and then at the same appointment injected into the origin of extensor carpi radialis brevis. The injection is often done using ultrasound visualization, and a one millilitre of lidocaine or marcaine is added to the injection.

Given the physiological theory behind injecting autologous blood into a degenerated tendon, postinjection therapy regimes would need to support the initial healing phase thought to occur following injection [6]. Four studies mentioned postinjection rest in a wrist support splint or sling; then normal activity was resumed by six weeks after injection. In two studies patients were told to perform only light duties or use modified lifting for up to four weeks after injection. Stretching exercises were named in two studies; apart from these, postinjection therapy was not described in detail.

There are eight published studies and one conference abstract of level II and IV evidence, of good to poor quality [7] regarding the efficacy of ABI in reducing pain in patients with tennis elbow. These studies (summarized in Table 1) varied in the chronicity of patients' symptoms, number of injections provided, type of assessments, and duration of followup of patients. The aim of this study is to evaluate the effect of ABI, splinting, and occupational therapy for patients with chronic LE, who have not been relieved by cortisone injection.

## 2. Materials and Methods

Approval for this study was obtained from the Ethics and Compliance Committee of the University of South Australia. All patients provided informed consent prior to participation in the study.

Patients had to be over the age of 18 years and able to provide informed consent. They had to have experienced lateral epicondylitis as diagnosed by a physician (AP) for a minimum of six months, and they had to have had at least one steroid injection before being offered autologous blood. Patients then underwent a diagnostic ultrasound to confirm their appropriateness for ABI; they had to show signs of tendon origin degeneration rather than inflammation, as diagnosed by one radiologist. Consecutive patients who fit these eligibility criteria were invited into the study.

Patients were fitted with a custom fabricated volar wrist resting splint in a neutral to 10° extension wrist position prior to injection. The patients then returned to their physician and had two millilitres of venous blood withdrawn from their contralateral arm, mixed with one millilitre of bupivacaine, which was then injected using ultrasound guidance into the tendinous origin of their extensor carpi radialis brevis tendon. Patients spent five days following injection resting in the custom made wrist splint and then were taught elbow, wrist, and hand range of motion, stretching dynamic concentric and later eccentric strengthening and self-monitoring techniques. Their performance of the exercises was checked fortnightly for the first four weeks by their occupational therapist and then as required for up to 12 weeks.

Short-term assessments, made at baseline, three andsix months following injection included visual analoguemeasures of pain (at worst, at best, and on average);grip strength, using a Jamar dynamometer and protocol from the American Society of Hand Therapists Clinical Assessment Recommendations [17].

Patients completed the Patient Rated Tennis Elbow Evaluation [18] (formerly the Patient Rated Forearm Questionnaire) before treatment, after six months and annually after that by telephone interview. This questionnaire has three subscales of questions asking patients to rate their pain, specific abilities, and overall function in numerical analogues. The worst possible score is 100 and the best is zero.

Power calculation based upon significant (*P* < 0.05) pain reduction of more than three points on a ten-point visual analogue scale suggested that thirty-two patients were needed for this study. All data were deidentified, and assessment scores of function, pain, and strength were evaluated for normalcy of distribution, and changes in all outcome measures were calculated.

## 3. Results

Forty-five consecutive patients were eligible for this study and 40 consented to participate; one patient had two elbows included in the study. There were initially 8 men and 32 women aged 33 to 56 years, mean 47 years (SD 5.7). Three patients were excluded during the study because they had other procedures such as carpal tunnel release and shoulder injections on the same arm, which altered their pain and function. Two further patients were eliminated because they had lateral epicondyle release after their ABI, and six declined to return for assessment as their symptoms were resolved, so there were 28 elbows in the final analysis. Eight patients had shoulder pain or wrist pain on the same side as their lateral epicondylitis.

The duration of patients' symptoms ranged from seven months to six years (CI 18 months to three years). Thirty-seven were under workers' compensation, and three were private patients. All had one or more cortisone injection, plus either electric shock wave therapy, ultrasound, NSAIDS, braces to the forearm and wrist, and exercise therapy without satisfactory relief from symptoms. Radiologic assessment of all included patients described degeneration or discontinuity in the tendinous origin of extensor carpi radialis brevis, some with calcification of the tendon.

No patients experienced severe bruising, and none had infection on the donor arm or on the injected arm. Significant improvements occurred in self-perceived measures of pain and upper limb function and in women's hand grip strength. Two of the eight men lost hand grip strength, one by five kilograms and the other by seven.  Table 2 shows 95% confidence intervals for pain and grip strength.

Patient-rated tennis elbow evaluation scores at baseline were (95%CI) 40–52/100. At 12 weeks they were 13–26/100. Between 18 months and 5 years, four patients were lost to followup. Remaining patients rated 13–27100, a significant decrease in pain and increase in function (*P* = 0.002).

## 4. Discussion

This study of chronic patients shows positive medium and long-term results from a single injection of autologous blood. The injection under ultrasound visualization accompanying treatments, splinting, and occupational therapy were free of adverse events such as severe bruising or infection.

This study was slightly different to past studies in patient selection; all were symptomatic for at least six months and had failed cortisone injection treatment. Radiology reports suggested degenerative changes and “discontinuity” or tears, in the tendinous origin at the lateral epicondyle. This suggests that these patients had a more degenerative than inflammatory condition, despite their diagnosis being lateral epicondylitis.

One limitation of this study was the absence of randomization into a control group. The reason for this was that most patients were under worker's compensation and the doctor treating them felt it unethical to deny the injection to those with chronic and resistant symptoms. A comparison group was initially formed but these patients were unlike those in the study, in their duration of symptoms.

More recent studies of ABI for lateral epicondylitis have injected plasma-rich proteins (PRPs) which have been injected in the same manner as untreated autologous blood. Preparation of PRP involves withdrawing approximately 27 millilitres of anticoagulated blood and placing it in a centrifuge, before adding anaesthetic and injecting [2]. Platelet-rich plasma has positive effects, but in many studies these results have not been significantly different to ABI results [10, 13].

Considerable thought was given to the issue of comorbidity. In this study, eight patients had diagnoses of carpal tunnel syndrome, shoulder overuse, ulnar neuropathy, and osteoarthritis of the wrist. Two patients were excluded from the follow-up analyses because they had surgery to their elbows, but other patients had procedures of carpal tunnel release, wrist fusion, and arthroscopic shoulder repair in the years following their elbow surgery. Although the symptoms from these conditions may affect the results of pain and functional evaluation, these results are representative of occupational overuse syndromes of the upper limbs [19, 20].

A further limitation of this study was that some patients declined to return to the clinic for their six-month assessment of grip function. These patients were under worker's compensation, and it was not reasonable to request that they return to occupational therapy in work hours. Intention to treat analysis was not used as this would likely show an inaccurate picture of their function.

When the patient-rated tennis elbow evaluation was completed with the occupational therapist, the patients' comments were recorded; numerous patients said that they had no functional limitations, scoring the highest marks on this evaluation. These patients also said that they now did things differently, had altered their work duties, or ceased certain activities. In one case, a woman changed her vacuum cleaner to a self-propelling model, and she had no pain or difficulty with vacuuming. These strategies were all successful but perhaps give more positive functional results than can be compared with the patients' original activities. Despite these limitations, this study still describes dramatic improvement in the functional ability of patients with chronic degenerative tennis elbow, who had autologous blood injection wrist immobilisation and a home exercise programme, including those under worker's compensation, and having upper limb comorbidities.

## Figures and Tables

**Table 1:** The literature regarding published outcomes of autologous blood injection for lateral epicondylitis. Units are as authors have provided, in means, confidence intervals (CI) or percentages.

Study and design	Participants	Intervention/comparison	Outcome measures	Result
Thanasas et al. [8] RCT	*N* = 28, symptom duration equal to or more than 3 months, no previous local injection treatment	*N* = 14 treated with autologous blood injection under ultrasound guidance *N* = 14 treated with platelet rich plasma under ultrasound guidance Postinjection eccentric strengthening followed by all	Pain visual analogue score, 6/12 Liverpool elbow score, 6/12	No significant difference between groups by Liverpool elbow score Mean visual analogue score improvement Platelet rich group; 3.8 points (95% confidence interval (CI), 3.1–4.5) Autologous blood injection group; 2.5 points (95% CI, 1.9–3.1)

Wolf et al. [9] RCT	*N* = 34 patients, 28 followed up <6/12 history of lateral epicondylitis	Injection of saline and lidocaine, or corticosteroid and lidocaine, or autologous blood and lidocaine rehabilitation regime not provided	Pain visual analogue score, Disabilities of the arm, shoulder and hand questionnaire and patient-rated Forearm Evaluation to 6/12	Patients in each injection group had significantly improved outcome scores at 6/12 Autologous blood or corticosteroid provided no advantage over placebo saline.

Creaney et al. [10] RCT	*N* = 150, patients who had physiotherapy treatment without improvement 130 followed up	Platelet rich plasma injection (*N* = 80) Autologous blood injection (*N* = 70) rehabilitation regime not provided	patient rated tennis elbow Evaluation to 6/12, need for surgery	43/60 autologous blood injection patients had at least 25/100 points improvement on the patient rated tennis elbow evaluation 12 autologous blood injection patients had surgery

Ozturan et al. 2010 [11] RCT	*N* = 60 patients ≥6/12 symptoms *N* = 20 in each treatment group	One-two cortisone injections or one-two autologous blood injections or three extracorporeal shock wave treatments rehabilitation regime not provided	Thomsen test, grip strength and ? self-designed functional scale at one year	At 4 weeks cortisone provided best result but at one year 50% relapse Best results at one year for extracorporeal shock wave and then autologous blood injection

Bharti et al. 2010 [1] case series	*N* = 25≤1/12 symptom duration	One autologous blood injection rehabilitation regime not provided	Pain visual analogue score Verhaar scoring system of pain, satisfaction subjective grip and Thomsen test of pain on resisted wrist extension to 12/52	One poor and one fair result, 23 good/excellent

Kazemi et al. 2010 [2] RCT	*N* = 60 patients *N* = 30 in each treatment group First sign of symptoms	One-two cortisone injections or one-two autologous blood injections. Patients told to return to activity gradually	Disabilities of the arm, shoulder, and hand questionnaire Nirschl Score Pain visual analogue score Grip Pain Algometer to 8/52	Significant improvements after both interventions, better results for autologous blood injection

Edwards and Calandruccio [6] case series	*N* = 28 who had no previous treatment for lateral epicondylitis uncertain duration of symptoms	One-three autologous blood injections 3/52 rest in wrist splint Return to normal activity at 6/52	Nirschl score Pain visual analogue score for 6/12 to 2 years	22/28 complete pain relief Significant improvement in all patients

Dehghani et al. [12] case series (abstract)	*N* = 22 who previously failed conservative treatment uncertain duration of symptoms	One autologous blood injections rehabilitation regime not provided	Pain visual analogue score 100 point scale patient rated tennis elbow Evaluation uncertain duration of followup	Average pain scores went from 44–9 points Average patient rated tennis elbow evaluation scores went from 42 to 10 points

Ul Gani et al. [13] case series	*N* = 26 3/12 to 2 years symptomatic Excluded if did not attend followup	One-two autologous blood injections Sling for 1/52 Light duties for 3/52 Wrist extensor stretches at 4/52	Nirschl score Pain visual analogue score for a mean of 8/12	Mean Nirschl score went from 3.3 to 1.2 Mean Pain score went from 5.5 to 3/10 10/26 satisfied 11/26 not satisfied 2nd injection of no greater benefit

Saldana [14] RCT Conference abstract	*N* = 75 with Pain visual analogue score >5/10 36 assigned to steroid injection 39 assigned to autologous blood	One Steroid injection = 9 Two Steroid injections = 14 Three Steroid injections = 3 OR One autologous blood and analgesic = 30 Two autologous blood and analgesic = 9 All patients advised to use nonsteroidal anti-inflammatories, grasp with forearm in supination, stretch, use splints	Pain visual analogue score uncertain duration of followup	10 healed with one injection of blood 12 healed with three injections of steroid

Connell et al. [15] case series	*N* = 35 symptoms for 6/12 to 4 years	Dry needling and autologous blood with ultrasound guidance 26 patients had two injections, three patients had three injections	Nirschl score Pain visual analogue score to 6/12	Significant improvement in all patients except two who had surgery

Wang et al. [16]	Unable to retrieve full paper			

**Table 2:** Shows the 95% CI for pain, grip, and the patient-rated tennis elbow Evaluations of the 38 patients before injection, 12 weeks after injection, 26 weeks and one to four years after injection.

Measure		Before injection	12 Weeks	26 Weeks
		*N* = 38	*N* = 38	*N* = 32
Visual analogue pain at worst 0–10		7.5–8.8	3.3 – 5	2.6–5.2*
Visual analogue pain at best 0–10		1.4–2.8	0.3–1.5	0.2–1.1*
Visual analogue average pain 0–10		3.6–4.9	2.2–3.7	0.9–2.1*
Hand Grip Strength (kilograms)	Men	37–53	40–56	42–59
	Women	18–23	20–26	23–29*

*significant at *P* < 0.000
